# Intradialytic blood pressure instability and associated factors among hemodialysis patients in Somalia: a retrospective study

**DOI:** 10.3389/fneph.2026.1822440

**Published:** 2026-06-23

**Authors:** Abdulrashid Hashi Mohamed, Mohamud Mire Waberi, Feyza AKSU, Nurto Abdulkadir Said, Said Abdirahman Ahmed

**Affiliations:** 1 Department of Internal Medicine, Mogadishu Somali Turkish Training and Research Hospital, Mogadishu, Somalia; 2 Faculty of Medicine and Surgery, Al Hayat Medical University, Mogadishu, Somalia; 3 Department of Cardiology, Mogadishu Somali Turkish Training and Research Hospital, Mogadishu, Somalia; 4 Department of Cardiology at Göztepe Prof. Dr. Süleyman Yalçın City Hospital, Istanbul, Türkiye; 5 Department of Public Health, Mogadishu University, Mogadishu, Somalia

**Keywords:** chronic kidney disease, hemodialysis, intradialytic blood pressure changes, intradialytic hypertension, intradialytic hypotension, ultrafiltration

## Abstract

**Background:**

Intradialytic blood pressure (BP) instability—manifesting as intradialytic hypotension (IDH) or hypertension (IDHT)—is increasingly recognized as a contributor to cardiovascular morbidity in hemodialysis patients. Despite its clinical significance, data on intradialytic BP behavior from sub-Saharan Africa remain scarce.

**Objective:**

To determine the prevalence and clinical factors associated with intradialytic BP changes among chronic kidney disease (CKD) patients on maintenance hemodialysis in a tertiary hospital in Somalia.

**Methods:**

We conducted a retrospective observational study of adult patients undergoing maintenance hemodialysis at Mogadishu Somali Turkish Training and Research Hospital between June and September 2022. Intradialytic hypotension was defined as a ≥20 mmHg drop in systolic BP and/or ≥10 mmHg drop in mean arterial pressure (MAP), while IDHT was defined as a >10 mmHg increase in systolic BP and/or ≥15 mmHg increase in MAP from pre- to post-dialysis. Demographic, clinical, dialysis, and laboratory variables were extracted. Logistic regression models were used to evaluate factors associated with IDH and IDHT, with reduced modeling for IDHT because of the small number of events.

**Results:**

Among 149 patients (mean age 51.1 ± 16.4 years; 57.7% female), the prevalence of IDH and IDHT was 55.0% and 12.1%, respectively. Most patients (74.5%) had hypertension and 96.5% used an arteriovenous fistula. In multivariable analysis, higher pre-dialysis systolic BP (OR 1.037; 95% CI 1.018–1.056) and ultrafiltration goal (OR 1.537; 95% CI 1.002–2.357) were associated with IDH. Because pre-dialysis systolic BP contributed to the outcome definition, this association should be interpreted cautiously. In the reduced IDHT model, ultrafiltration goal was inversely associated with IDHT (OR 0.543; 95% CI 0.298–0.990), while antihypertensive medication burden was not statistically significant. Exploratory electrolyte tertile analysis showed a higher proportion of IDH among patients in lower calcium and higher phosphate tertiles.

**Conclusion:**

Intradialytic BP instability was common among hemodialysis patients in Somalia, particularly IDH. Given the retrospective design, reliance on pre- and post-dialysis BP measurements, and limited number of IDHT events, the findings should be interpreted as associative and hypothesis-generating. Routine BP monitoring and further prospective studies are warranted to better characterize hemodynamic instability in resource-constrained dialysis settings.

## Introduction

Chronic kidney disease (CKD) is a major and expanding global health problem, with a rising proportion of patients progressing to end-stage renal disease (ESRD) requiring renal replacement therapy (1, 2). Hemodialysis remains the dominant treatment modality worldwide, particularly in low- and middle-income countries where access to kidney transplantation and peritoneal dialysis is limited by economic and infrastructural constraints (3, 4). While hemodialysis is life-sustaining, it exposes patients to repetitive hemodynamic stress, most notably intradialytic blood pressure (BP) instability, which has emerged as a key contributor to cardiovascular morbidity and mortality (5, 6).

Intradialytic hypotension (IDH) and intradialytic hypertension (IDHT) are no longer regarded as benign procedural phenomena but as clinically meaningful disorders with prognostic implications (7, 8). In this study, IDH was operationalized as a decrease in systolic BP ≥20 mmHg and/or a decrease in mean arterial pressure ≥10 mmHg from pre- to post-dialysis based on routinely recorded dialysis-unit measurements (9). Depending on patient characteristics, dialysis setting, and definitions applied, intradialytic hypotension remains common among patients receiving hemodialysis, with recent pooled evidence confirming a substantial burden of cardiovascular instability during dialysis therapy (10, 11). Recurrent IDH has been associated with myocardial stunning, cerebral hypoperfusion, vascular access thrombosis, accelerated loss of residual kidney function, and increased all-cause mortality (12, 13).

In contrast, IDHT—characterized by a paradoxical rise in BP during or immediately after hemodialysis—affects approximately 5–15% of patients receiving maintenance hemodialysis (14, 15). IDHT has been consistently linked to adverse cardiovascular outcomes, including left ventricular hypertrophy, heart failure, increased hospitalization, and mortality (16, 17). Proposed mechanisms include persistent volume overload, endothelial dysfunction, heightened sympathetic activity, activation of the renin–angiotensin–aldosterone system (RAAS), and increased arterial stiffness (18, 19).

Hypertension remains highly prevalent among patients with ESRD, affecting more than 80% of individuals on maintenance hemodialysis (20). However, BP behavior during dialysis often diverges from interdialytic BP control, underscoring the complex interplay between ultrafiltration, vascular tone, neurohormonal activation, and limited cardiac reserve. Increasingly, dynamic intradialytic BP patterns are being recognized as superior prognostic markers compared with isolated pre- or post-dialysis BP measurements.

Despite growing interest in intradialytic BP abnormalities, most existing evidence originates from high-income countries with standardized dialysis protocols and broad access to advanced monitoring and pharmacologic interventions. Data from resource-limited settings, particularly in sub-Saharan Africa, remain scarce. Understanding intradialytic BP behavior in such contexts is essential for developing pragmatic, context-appropriate strategies to mitigate cardiovascular risk.

Accordingly, this study aimed to evaluate the prevalence and determinants of intradialytic blood pressure changes among CKD patients undergoing maintenance hemodialysis at a tertiary hospital in Mogadishu, Somalia, and to examine their associations with demographic, clinical, and dialysis-related factors. We hypothesized that intradialytic blood pressure instability would be highly prevalent in this setting due to advanced disease presentation and limited access to individualized dialysis protocols.

## Methods

### Study design and setting

We conducted a retrospective observational cohort study at Mogadishu Somali Turkish Training and Research Hospital (Mogadishu, Somalia), a tertiary referral center providing maintenance hemodialysis services. The study period covered hemodialysis sessions between June 1, 2022 and September 30, 2022. This study was reported in accordance with the Strengthening the Reporting of Observational Studies in Epidemiology (STROBE) guidelines for cohort studies (21).

### Participants

Eligible participants were adults (≥18 years) receiving maintenance intermittent hemodialysis at the dialysis unit during the study period. A consecutive sampling strategy was used, whereby all eligible patients with available records during the study period were included. We included patients with recorded pre- and post-dialysis blood pressure measurements and sufficient clinical and dialysis prescription data for analysis. Patients with fewer than three recorded hemodialysis sessions in the source records or missing key blood pressure measurements required for outcome classification were excluded from the complete-case regression analyses. For each patient, blood pressure measurements from three consecutive recorded dialysis sessions were averaged to minimize intra-individual variability, and all statistical analyses were conducted at the patient level.

### Data sources and measurement

Data were abstracted from routinely collected hospital dialysis records and medical files. Variables extracted included demographics (age, sex, weight), comorbidity category (no comorbidity, hypertension, diabetes mellitus, diabetes plus hypertension), vascular access type (arteriovenous fistula or catheter), dialysis prescription parameters (blood flow rate, ultrafiltration goal, dialysis duration, number of sessions), laboratory parameters (sodium, potassium, calcium, phosphate, urea, creatinine, hematocrit, estimated glomerular filtration rate), antihypertensive medication burden (number of prescribed antihypertensive agents), and pre- and post-dialysis systolic and diastolic blood pressure. Mean arterial pressure (MAP) was calculated as MAP = DBP + (SBP − DBP)/3. Blood pressure measurements were obtained as part of routine dialysis care immediately before dialysis initiation and immediately after completion of dialysis. Because of the retrospective design, detailed information regarding patient positioning and full measurement standardization was not consistently available.

### Dry weight assessment

Dry weight in our dialysis unit was determined clinically by the treating nephrologist based on interdialytic weight gain, blood pressure trends, presence or absence of peripheral edema, intradialytic symptoms, and post-dialysis clinical assessment. Objective tools such as bioimpedance analysis, BNP/NT-proBNP measurement, or routine echocardiography were not available during the study period. Dry weight was reassessed periodically based on clinical judgment and patient tolerance to ultrafiltration.

### Outcomes

The primary outcomes were intradialytic hypotension (IDH) and intradialytic hypertension (IDHT), operationalized from routinely recorded pre- and post-dialysis blood pressure values.

- IDH: a decrease in systolic blood pressure ≥20 mmHg and/or a decrease in MAP ≥10 mmHg from pre- to post-dialysis.
- IDHT: an increase in systolic blood pressure >10 mmHg and/or an increase in MAP ≥15 mmHg from pre- to post-dialysis (14).

Patients who met predefined criteria for intradialytic hypotension or intradialytic hypertension were classified accordingly. Patients who did not meet criteria for either outcome were classified as having a stable intradialytic BP pattern. No patients met criteria for both intradialytic hypotension and intradialytic hypertension after application of the predefined outcome definitions.

### Bias and confounding

As a retrospective study, the analysis was susceptible to information bias (measurement and recording variability) and residual confounding. To mitigate confounding, we pre-specified clinically plausible covariates and used multivariable regression with parsimonious models to avoid overfitting, particularly for IDHT where events were limited.

### Study size

The sample size was determined by all eligible patients available in the study period dataset. No *a priori* sample size calculation was performed due to the retrospective design.

### Statistical analysis

Continuous variables are summarized as mean ± standard deviation (SD) and categorical variables as counts and percentages. Between-group comparisons were performed using chi-square tests for categorical variables and ANOVA for continuous variables, as appropriate. Associations between calcium and phosphate tertiles and intradialytic blood pressure patterns were evaluated using chi-square tests for trend.

For re-analysis, we fit separate multivariable logistic regression models for:

1. IDH vs. non-IDH, and
2. IDHT vs. non-IDHT.

Predictors were selected *a priori* and constrained by the number of outcome events (especially for IDHT). For IDH, we included age, sex, ultrafiltration goal, pre-dialysis systolic BP, comorbidity category, and antihypertensive medication count. Catheter use was not included in the final IDH model because only five patients had catheter access, producing unstable estimates with wide confidence intervals. For IDHT, given the small number of outcome events, we used a reduced parsimonious model including ultrafiltration goal, antihypertensive medication count, and comorbidity category. Catheter use was not included in the IDHT model because only five patients had catheter access, which would produce unstable estimates.

Multicollinearity among independent variables was assessed using variance inflation factors (VIF), with values >5 considered suggestive of potentially problematic collinearity. Model discrimination was evaluated using the C-statistic/area under the receiver operating characteristic curve (AUC). Model calibration was assessed using the Hosmer–Lemeshow goodness-of-fit test. Overall model performance was further evaluated using McFadden’s pseudo-R² and likelihood ratio testing. Model diagnostics were performed by inspecting deviance residuals plotted against predicted probabilities to identify potentially influential observations and assess overall model fit.

### Ethics approval and consent to participate

Ethical approval for this study was obtained from the Institutional Review Board of Mogadishu Somali Turkish Training and Research Hospital (Reference: MSTH/**23474**). The study complied with the ethical principles of the Declaration of Helsinki. As this was a retrospective review of de-identified clinical records, the requirement for individual informed consent was waived.

## Results

### Patient characteristics

A total of 149 patients with chronic kidney disease undergoing maintenance hemodialysis were included in the analysis. The mean age was 51.1 ± 16.4 years, and 57.7% of the participants were female. Regarding comorbidities, hypertension was present in 111 patients (74.5%) and diabetes mellitus in 21 patients (14.1%). For regression modeling, comorbidity was analyzed using mutually exclusive categories: no comorbidity, hypertension only, diabetes mellitus only, and hypertension plus diabetes mellitus. The majority of patients (96.5%) utilized an arteriovenous fistula as their vascular access. With respect to intradialytic blood pressure (BP) patterns, intradialytic hypotension (IDH) occurred in 82 patients (55.0%), while 49 patients (32.9%) maintained stable BP throughout dialysis sessions. Intradialytic hypertension (IDHT) was observed in 18 patients, accounting for 12.1% of the cohort (**Table 1**). The mean ultrafiltration goal was 2.42 ± 0.88 L. Mean pre-dialysis systolic blood pressure was 160.1 ± 29.4 mmHg, decreasing to 145.7 ± 29.8 mmHg post-dialysis.

**Table 1:** Baseline demographic and clinical characteristics stratified by intradialytic hypotension status.

Characteristic	Total (n=149)	IDH (n=82)	No IDH (n=67)	p-value
Age, mean ± SD (years)	51.0 ± 16.7	49.9 ± 17.1	52.4 ± 16.1	0.357
Female sex, n (%)	85 (57.0)	47 (57.3)	38 (56.7)	0.941
Weight, mean ± SD (kg)	59.1 ± 12.5	58.7 ± 12.5	59.6 ± 12.6	0.659
Hypertension, n (%)	111 (74.5)	58 (70.7)	53 (79.1)	0.243
Diabetes mellitus, n (%)	21 (14.1)	11 (13.4)	10 (14.9)	0.792
AV fistula, n (%)	144 (96.6)	79 (96.3)	65 (97.0)	1.000
Catheter access, n (%)	5 (3.4)	3 (3.7)	2 (3.0)	1.000
Ultrafiltration goal, mean ± SD (L)	2.4 ± 0.9	2.6 ± 0.8	2.2 ± 0.9	0.023
Pre-dialysis SBP, mean ± SD (mmHg)	160.1 ± 29.4	165.7 ± 30.8	153.2 ± 26.2	0.008
Post-dialysis SBP, mean ± SD (mmHg)	145.7 ± 29.8	136.1 ± 27.4	157.5 ± 28.4	<0.001
No. of antihypertensives, mean ± SD	1.0 ± 0.8	0.9 ± 0.7	1.0 ± 0.8	0.327
Serum calcium, mean ± SD (mmol/L)	8.3 ± 1.0	8.2 ± 1.1	8.5 ± 1.0	0.038
Serum phosphate, mean ± SD (mmol/L)	3.5 ± 1.9	3.7 ± 2.1	3.2 ± 1.6	0.073

Values are presented as mean ± SD or n (%). IDH, intradialytic hypotension; SBP, systolic blood pressure; SD, standard deviation.

### Multivariable logistic regression analysis

Multivariable logistic regression was performed to evaluate factors associated with intradialytic hypotension and intradialytic hypertension. After adjustment for relevant covariates, a higher ultrafiltration goal and elevated pre-dialysis systolic blood pressure were associated with increased odds of intradialytic hypotension (**Table 2**). Because only 18 patients experienced intradialytic hypertension, the IDHT model was reduced and interpreted cautiously. In this reduced model, ultrafiltration goal was inversely associated with IDHT, while antihypertensive medication burden and comorbidity categories were not statistically significant (**Table 3**). Given the small number of IDHT events, these findings should be considered exploratory rather than confirmatory.

**Table 2:** Univariable and final multivariable logistic regression analysis of factors associated with intradialytic hypotension.

Predictor	Unadjusted OR	95% CI	p-value	Adjusted OR	95% CI	p-value
Pre-dialysis SBP, per 1 mmHg increase	1.015	1.003–1.027	0.011	1.037	1.018–1.056	<0.001
Ultrafiltration goal, per 1 L increase	1.565	1.060–2.311	0.024	1.537	1.002–2.357	0.049
No. of antihypertensives, per 1 medication increase	0.809	0.533–1.229	0.320	0.617	0.335–1.137	0.121
Age, per 1-year increase	0.991	0.972–1.010	0.358	0.988	0.966–1.010	0.273
Female sex (reference: male)	1.025	0.534–1.967	0.941	1.380	0.653–2.918	0.399

OR, odds ratio; CI, confidence interval; SBP, systolic blood pressure. Adjusted model included age, sex, ultrafiltration goal, pre-dialysis systolic BP, comorbidity category, and antihypertensive medication count. Catheter access was excluded from the final IDH model because only five patients had catheter access, resulting in unstable estimates.

**Table 3:** Multivariable logistic regression analysis of predictors of intradialytic hypertension.

Predictor	Adjusted OR	95% CI	p-value
Ultrafiltration goal, per 1 L increase	0.543	0.298–0.990	0.046
No. of antihypertensives, per 1 medication increase	1.801	0.831–3.901	0.136
Hypertension only (reference: no comorbidity)	0.909	0.178–4.638	0.908
Diabetes mellitus only (reference: no comorbidity)	2.782	0.222–34.882	0.428
Hypertension + diabetes (reference: no comorbidity)	1.141	0.141–9.215	0.902

OR, odds ratio; CI, confidence interval. The reduced IDHT model excluded pre-dialysis systolic blood pressure because it contributed to the outcome definition and excluded catheter access because only five patients had catheter access, which could produce unstable estimates. IDHT results should be interpreted cautiously because of the small number of outcome events.

Pre-dialysis systolic blood pressure should be interpreted cautiously as a predictor because it also contributes to the outcome definition, introducing the possibility of mathematical coupling and regression-to-the-mean effects. Consequently, the observed associations may partially reflect statistical artifact rather than a true physiological relationship.

### Biochemical stratification of intradialytic blood pressure changes

**Figure 1:**
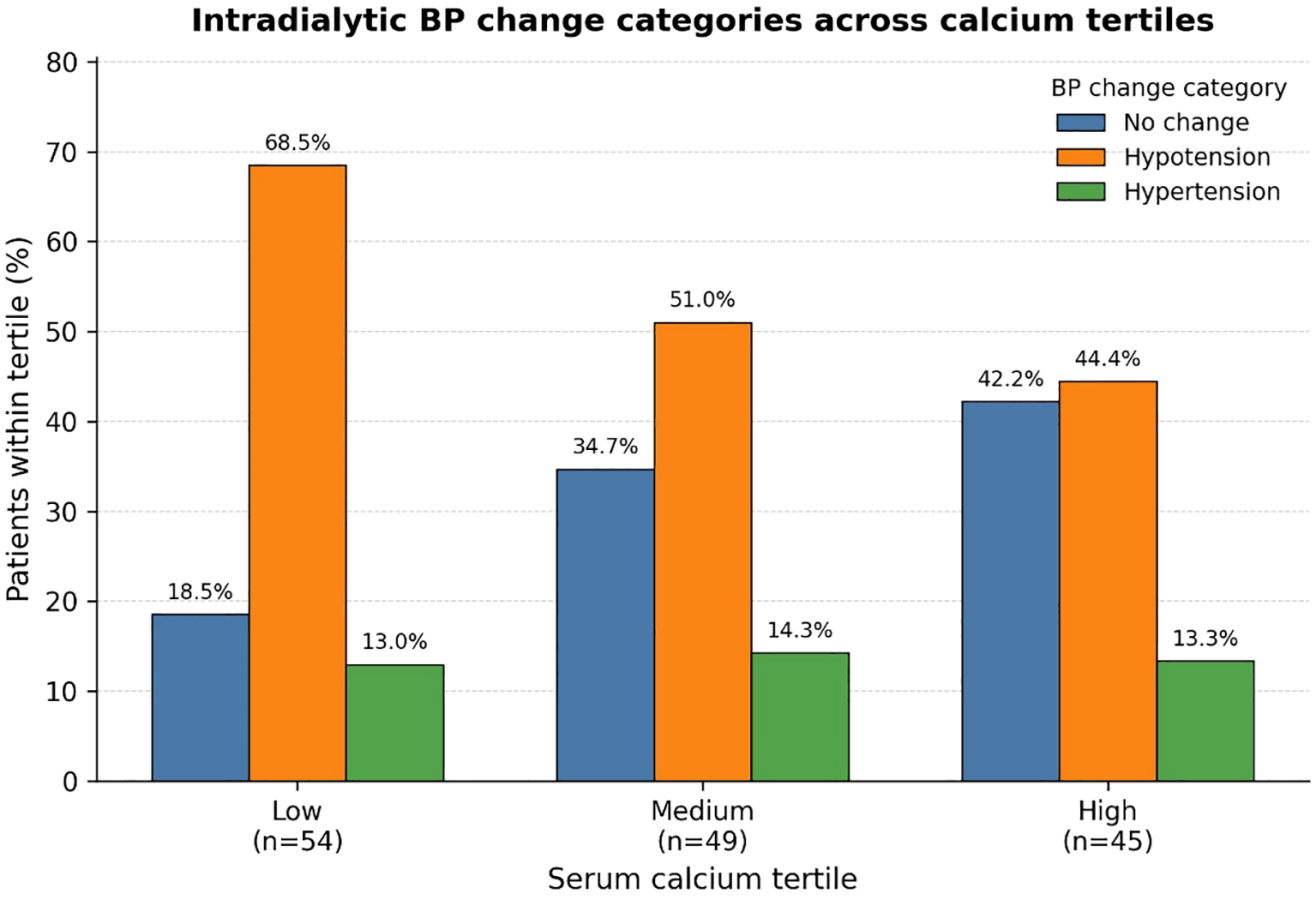
Distribution of intradialytic blood pressure change categories across calcium tertiles (n = 149).

Intradialytic blood pressure patterns were examined across tertiles of serum calcium and phosphate levels using within-tertile proportions. The proportion of patients with intradialytic hypotension was highest in the lowest calcium tertile and decreased across increasing calcium tertiles (low: 37/54, 68.5%; middle: 25/49, 51.0%; high: 20/45, 44.4%; p for trend = 0.02). No significant association was observed between calcium tertiles and intradialytic hypertension (low: 7/54, 13.0%; middle: 7/49, 14.3%; high: 6/45, 13.3%; p > 0.80) (**Figure 1**).

**Figure 2:**
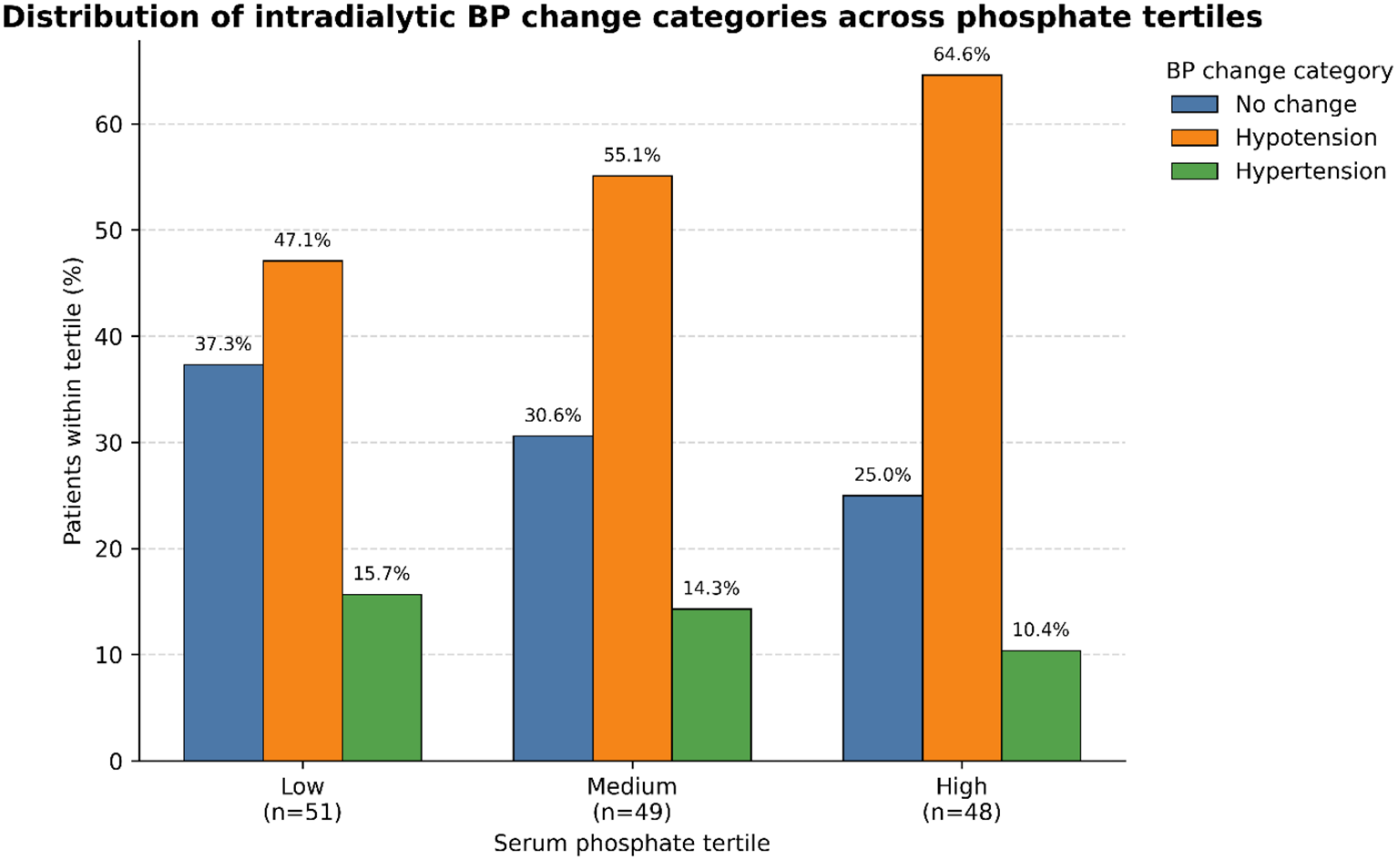
Distribution of intradialytic blood pressure change categories across phosphate tertiles.

For phosphate, the proportion of patients with intradialytic hypotension increased across tertiles (low: 24/51, 47.1%; middle: 27/49, 55.1%; high: 31/48, 64.6%; p for trend = 0.04). No significant association was observed between phosphate tertiles and intradialytic hypertension (low: 8/51, 15.7%; middle: 7/49, 14.3%; high: 5/48, 10.4%; p > 0.50) (**Figure 2**). Because these analyses were exploratory and unadjusted, the findings should be interpreted cautiously.

### Model diagnostics and performance

No problematic multicollinearity was identified in either model, with all VIF values below 5. The IDH model demonstrated acceptable discrimination (AUC = 0.738) and adequate calibration by the Hosmer–Lemeshow test (χ² = 11.30, df = 8, p = 0.185). The final IDH model showed improved fit compared with the null model (likelihood ratio χ² = 26.09, df = 8, p = 0.0010; McFadden’s pseudo-R² = 0.128). Inspection of deviance residuals did not identify influential observations requiring exclusion (**Table 4**).

**Table 4:** Logistic regression model diagnostics and performance.

Diagnostic	IDH model	IDHT model
Complete-case sample size	148	148
Outcome events	82	17
Maximum VIF	2.51	<5
AUC/C-statistic	0.738	0.784
Hosmer–Lemeshow χ² (df)	11.30 (8)	5.50 (8)
Hosmer–Lemeshow p-value	0.185	0.703
Likelihood ratio χ² (df)	26.09 (8)	14.45 (7)
Likelihood ratio p-value	0.0010	0.044
McFadden’s pseudo-R²	0.128	0.137
Deviance residual findings	No observations with absolute deviance residuals >2	6 observations with |residual| >2 retained after review

One patient with intradialytic hypertension was excluded from the complete-case regression analyses because of missing covariate data. Consequently, the regression diagnostic sample included 148 patients and 17 IDHT events, whereas the overall study cohort included 149 patients and 18 IDHT events.

The IDHT model demonstrated acceptable discrimination (AUC = 0.784) and adequate calibration (Hosmer–Lemeshow χ² = 5.50, df = 8, p = 0.703). The final IDHT model showed improved fit compared with the null model (likelihood ratio χ² = 14.45, df = 7, p = 0.044; McFadden’s pseudo-R² = 0.137). Six observations had absolute deviance residuals greater than 2; however, given the retrospective design and small number of IDHT events, these were retained to avoid data-driven exclusion (**Table 4**).

## Discussion

This study demonstrates a substantial burden of intradialytic blood pressure instability among patients undergoing maintenance hemodialysis in Mogadishu, Somalia. More than half of the cohort experienced intradialytic hypotension, while intradialytic hypertension was observed in approximately one-tenth of patients. These findings highlight the magnitude of intradialytic hemodynamic stress in this population and emphasize its clinical relevance in resource-limited dialysis settings.

Accordingly, the reported prevalence estimates should be interpreted as reflecting intradialytic blood pressure instability based on routinely recorded pre- and post-dialysis blood pressure changes rather than strictly KDIGO-defined intradialytic hypotension or hypertension.

The observed prevalence of intradialytic hypotension (55%) exceeds that reported in many cohorts from high-income countries and likely reflects a combination of advanced disease at presentation, limited cardiovascular reserve, and restricted availability of individualized ultrafiltration strategies (22, 23). In settings where bioimpedance-guided volume assessment and continuous hemodynamic monitoring are unavailable, ultrafiltration targets are often based on clinical judgment alone, increasing the risk of excessive intravascular volume depletion.

A reduction in post-dialysis blood pressure may in some patients represent physiological volume correction following ultrafiltration rather than pathological intradialytic hypotension. Therefore, the high IDH prevalence observed in this study should be interpreted cautiously in the absence of symptom data, nadir blood pressure values, and intervention records.

Unlike dialysis programs in high-income countries where thrice-weekly sessions, sodium profiling, and bioimpedance-guided volume assessment are routine, dialysis delivery in our setting relied entirely on clinical volume estimation and fixed dialysate composition. These structural differences may explain the higher IDH prevalence observed in our cohort compared with rates of 15–30% reported in standardized programs.

In the multivariable model, higher pre-dialysis systolic BP was associated with intradialytic hypotension. However, because IDH was defined using the pre-to-post dialysis BP change, this association should be interpreted cautiously due to potential mathematical coupling and regression-to-the-mean effects. Therefore, the finding may partly reflect the greater opportunity for BP decline among patients starting dialysis with higher systolic BP rather than a direct physiological predictor. Repeated intradialytic hypotension remains clinically relevant because it may contribute to myocardial ischemia and ventricular stunning, thereby creating a self-perpetuating cycle of hemodynamic intolerance and cardiovascular injury (24, 25).

Intradialytic hypertension, although less frequent, remains clinically important because previous studies have linked it to adverse cardiovascular outcomes. Potential mechanisms include persistent volume overload, sodium imbalance, endothelial dysfunction, heightened sympathetic activity, and increased arterial stiffness, particularly in settings lacking sodium profiling and objective volume assessment tools (26, 27).

Because only 18 patients experienced intradialytic hypertension, the IDHT analysis was intentionally reduced and should be interpreted cautiously. In the reduced model, ultrafiltration goal was inversely associated with IDHT, while antihypertensive medication burden and comorbidity categories were not statistically significant. Given the small number of IDHT events, these findings should be considered exploratory rather than confirmatory.

Clinically, these findings reinforce the importance of individualized dialysis prescriptions that incorporate baseline BP, comorbidity burden, and intradialytic BP behavior. Our findings suggest that patients with elevated pre-dialysis systolic blood pressure may represent a subgroup warranting closer hemodynamic monitoring and consideration of more conservative ultrafiltration strategies, particularly in resource-limited settings. Conservative ultrafiltration rates, frequent reassessment of dry weight, careful timing of antihypertensive medications, and enhanced intradialytic BP surveillance may represent pragmatic strategies to mitigate cardiovascular risk in similar settings (28, 29).

As dialysis services expand across low- and middle-income countries, the patterns observed in this study are likely to be replicated elsewhere. Incorporating routine assessment of intradialytic BP patterns into standard care pathways may offer a feasible, low-cost approach to cardiovascular risk stratification and outcome improvement in resource-constrained environments (23, 24).

Exploratory tertile analyses showed that lower serum calcium and higher serum phosphate were associated with a greater proportion of intradialytic hypotension, whereas no clear association was observed with intradialytic hypertension. These findings were supported by formal trend testing but should be interpreted cautiously because the analyses were unadjusted, exploratory, and not corrected for multiple comparisons. Disturbances in mineral metabolism may plausibly influence vascular reactivity and intradialytic hemodynamic tolerance, as calcium–phosphate abnormalities are linked to vascular smooth muscle contractility, arterial compliance, vascular calcification, and arterial stiffness (30–32, 33). Given the retrospective design and the absence of parathyroid hormone, dialysate calcium, and dialysis adequacy data, these findings should be viewed as associative and hypothesis-generating rather than causal.

### Study strengths and limitations

This study provides original data on intradialytic blood pressure patterns from a severely understudied hemodialysis population in Somalia. However, several limitations should be acknowledged. First, the definitions of IDH and IDHT were based on pre- and post-dialysis blood pressure measurements and therefore do not fully align with contemporary KDIGO recommendations, which incorporate symptoms, intradialytic nadir blood pressure, and dialysis-related interventions. Consequently, our definitions may have captured intradialytic blood pressure fluctuations rather than all clinically significant intradialytic hypotensive or hypertensive events. Because only pre- and post-dialysis blood pressure values were available, true intradialytic trajectories, including nadir or peak blood pressure during the dialysis session, could not be captured. Transient hypotensive or hypertensive episodes occurring mid-session with recovery before the end of dialysis may therefore have been misclassified. The retrospective design also limited our ability to verify uniform measurement conditions, including patient positioning and exact timing of blood pressure assessments. The retrospective design limits causal inference. Objective measures of volume status, such as NT-proBNP or hANP, bioimpedance, and echocardiographic parameters, were not routinely available. Interdialytic weight gain and antihypertensive medication timing could not be reliably assessed. Information regarding antihypertensive drug classes, dialyzability, pharmacokinetic properties, and timing of administration was not consistently available; therefore, residual confounding related to antihypertensive treatment patterns cannot be excluded. Data regarding intradialytic interventions, including saline administration, ultrafiltration adjustments, and medication administration during dialysis sessions, were also not consistently available and could not be incorporated into the analysis. Because pre-dialysis systolic blood pressure contributed to the operational definition of intradialytic blood pressure change, associations involving pre-dialysis systolic blood pressure may be affected by mathematical coupling and regression-to-the-mean. In addition, the relatively small number of intradialytic hypertension events required reduced parsimonious multivariable modeling, and IDHT-related findings should be interpreted as exploratory. These limitations should be considered when interpreting the findings.

This retrospective study evaluated intradialytic blood pressure patterns among patients receiving maintenance hemodialysis in a tertiary center in Mogadishu, Somalia. Intradialytic blood pressure instability was common, with intradialytic hypotension occurring in more than half of patients and intradialytic hypertension in a smaller but clinically important proportion. These patterns were associated primarily with baseline blood pressure and dialysis-related factors, highlighting the influence of hemodynamic and volume-related mechanisms on intradialytic blood pressure responses. The findings underscore the clinical relevance of monitoring intradialytic blood pressure behavior, particularly in resource-limited settings where patients often present late and advanced monitoring tools are unavailable.

## Conclusion

Intradialytic blood pressure instability was common among patients undergoing maintenance hemodialysis in Mogadishu, Somalia, with both hypotensive and hypertensive responses observed in routine practice. Because the study relied on pre- and post-dialysis blood pressure measurements rather than intradialytic nadir values, symptom documentation, or intervention records, these findings should be interpreted as descriptive of hemodynamic fluctuations rather than definitive KDIGO-defined clinical events. Incorporating systematic assessment of intradialytic blood pressure patterns into routine care may provide a pragmatic, low-cost approach to cardiovascular risk stratification in resource-limited settings. Prospective, multicenter studies using standardized intradialytic blood pressure trajectories, symptom assessment, intervention data, and detailed medication information are warranted to confirm these findings and inform targeted interventions.

## Data Availability

The original contributions presented in the study are included in the article/supplementary material. Further inquiries can be directed to the corresponding author.
