# Correction: Intradialytic blood pressure instability and associated factors among hemodialysis patients in Somalia: a retrospective study

**DOI:** 10.3389/fneph.2026.1921166

**Published:** 2026-07-07

**Authors:** Abdulrashid Hashi Mohamed, Mohamud Mire Waberi, Feyza Aksu, Nurto Abdulkadir Said, Said Abdirahman Ahmed

**Affiliations:** 1 Department of Internal Medicine, Mogadishu Somali Turkish Training and Research Hospital, Mogadishu, Somalia; 2 Faculty of Medicine and Surgery, Al Hayat Medical University, Mogadishu, Somalia; 3 Department of Cardiology, Mogadishu Somali Turkish Training and Research Hospital, Mogadishu, Somalia; 4 Department of Cardiology at Göztepe Prof. Dr. Süleyman Yalçın City Hospital, Istanbul, Türkiye; 5 Department of Public Health, Mogadishu University, Mogadishu, Somalia

**Keywords:** chronic kidney disease, hemodialysis, intradialytic blood pressure changes, intradialytic hypertension, intradialytic hypotension, ultrafiltration

In the published article, affiliation 4 was erroneously given as “Department of Nephrology at Göztepe Prof. Dr. Süleyman Yalçın City Hospital, Istanbul, Türkiye.”

The correct affiliation is “Department of Cardiology at Göztepe Prof. Dr. Süleyman Yalçın City Hospital, Istanbul, Türkiye.”

The original version of this article has been updated.

